# The Costs of a Multisectoral Nutrition Program Implemented Through a Poultry Value Chain Platform in Burkina Faso

**DOI:** 10.1111/mcn.13791

**Published:** 2025-01-03

**Authors:** Amy Margolies, Abdoulaye Pedehombga, Aisha Twalibu, Odiche Nwabuikwu, Jolene Wun, Chris Kemp, Aulo Gelli, Carol Levin

**Affiliations:** ^1^ International Food Policy Research Institute Washington District of Columbia USA; ^2^ AfricSanté Ouagadougou Burkina Faso; ^3^ Department of Global Health University of Washington Seattle Washington USA; ^4^ United States Department of International Development (USAID) Washington District of Columbia USA; ^5^ Johns Hopkins Bloomberg School of Public Health Baltimore Maryland USA

**Keywords:** agriculture, Burkina Faso, cost analysis, economic evaluation, gender, malnutrition, poultry

## Abstract

Undernutrition in women and young children in Burkina Faso is a critical problem. Egg consumption is low despite many households raising poultry. The Soutenir l'Exploitation Familiale pour Lancer l'Élevage des Volailles et Valoriser l'Économie Rurale (SELEVER) project, an integrated agriculture‐nutrition intervention, promoted egg consumption and sales to investigate the impact of poultry production on child nutrition. Multisectoral nutrition‐sensitive agriculture programs address nutrition deficits but lack comparable cost information. This study estimates the costs of the SELEVER program, an integrated poultry and nutrition intervention. The study estimates the program's economic costs using a standardized methodology from the Strengthening Economic Evaluation for Multisectoral Strategies for Nutrition (SEEMS‐Nutrition) consortium, which aligns financial and economic costs along program impact pathways, allocating costs by activities and inputs. We conducted qualitative interviews and focus groups on time allocation and beneficiary out‐of‐pocket costs. Incremental economic costs were calculated by combining expenditures and economic costs. The total incremental program cost was USD$18,084,727.68 over 5 years, with annual incremental costs of USD$209.20 per direct beneficiary and $796.26 per household. Major cost drivers included overhead (18%), poultry extension (17%), training (16%), household counseling (7%), technical assistance (7%) and microcredit (6%). Total input costs were dominated by personnel (51%), supplies (13%), agricultural inputs (10%) and overhead (9%). We present the total incremental costs of a multisectoral nutrition intervention to generate revenue with poultry. The costs per beneficiary were higher than similar interventions, underscoring the need for cost‐effectiveness evaluations of multisectoral nutrition programs. A standardized cost methodology facilitates comparisons with multisectoral nutrition interventions and builds the evidence base.

## Summary

- Despite their potential to improve the intake of nutritious foods, the costs of integrated livestock and nutrition programs remain unknown.
- The total costs per beneficiary of an integrated poultry, gender and nutrition program in Burkina Faso were on the higher end compared to similar interventions, with economic costs accounting for over 40% of of the total.
- Programs relying on volunteers—like many multisectoral behavior change interventions—should consider the opportunity costs of their time in design and implementation in order to improve sustainability.
- Combining our findings with the program's impact evaluation suggests that implementing a co‐location model—without fully integrating livestock, nutrition and gender dimensions—may not be either cost‐efficient or cost‐effective.

## Graphical abstract

Using a standardized costing approach developed for multisectoral nutrition interventions, the cost per participant of a co‐located poultry, gender and nutrition program were in the upper range of similar interventions. Economic costs accounted for 40% of total costs due to the significant opportunity costs of volunteers and participants, raising concerns for longer‐term uptake and sustainability of the intervention.

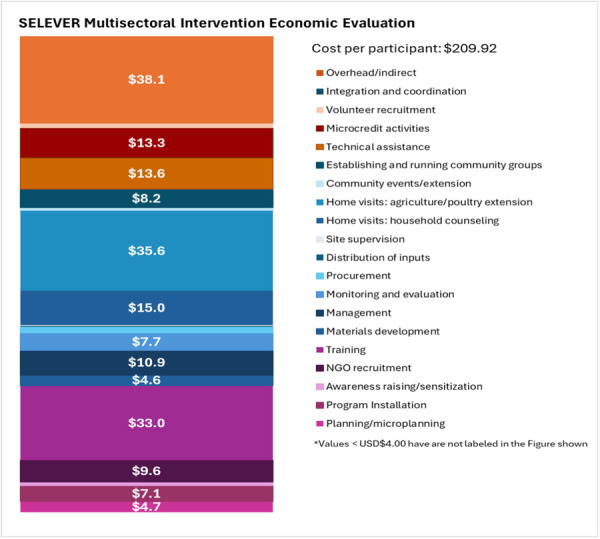

## 1 Background

Malnutrition in young children and their mothers is a persistent problem in Burkina Faso. Twenty‐three percent of children aged under 5 years are stunted, wasting affects 1a1%, and 18% are underweight (Burkina Faso DHS 2012). Twelve percent of women suffer from chronic energy deficiency(body mass index (BMI) below the normal threshold) and a growing proportion (21%)are classified as overweight (BMI > 25) (Burkina Faso DHS 2012).

Addressing undernutrition in low‐ and middle‐income countries (LMICs) involves scaling‐up nutrition‐specific interventions that tackle the immediate causes of malnutrition as well as nutrition‐sensitive interventions that target both intermediate and underlying causes. Nutrition‐sensitive programs often involve multisectoral platforms (preschools, microcredit, etc.) to implement activities to improve nutrition. Nutrition‐sensitive agriculture programs integrate behavior change communication (BCC) with actions to improve and diversify food production, such as agricultural extension and training. In countries with large agrarian populations, nutrition‐sensitive programs that incorporate agriculture show promise in increasing production of nutritious foods, diversifying maternal and child diets, and improving nutrition knowledge particularly on infant and young children feeding (IYCF) (Ruel et al. 2018). Interventions also incorporate gender sensitization and other actions to promote women's empowerment. Involving women in agriculture and nutrition has the potential to increase their decision‐making power and control of assets. That said, women's engagement in agriculture must not come at the cost of additional workloads or negative trade‐offs in time use or nutrition (Johnston et al. 2018; Margolies et al. 2023; van den Bold et al. 2021).

The agriculture components of these programs typically encourage diversification and nutritious food production. Increasingly, implementers and donors have focused on the potential of livestock and/or poultry or egg production as a feasible means by which to achieve agricultural and nutrition goals (Jodlowski et al. 2016; Kafle, Winter‐Nelson, and Goldsmith 2016; Lutter 2020; Morris, Beesabathuni, and Headey 2018; Phadera et al. 2019; Rawlins et al. 2014). Livestock‐derived foods are micronutrient‐rich and have the potential to improve maternal and child diets particularly during critical periods such as the first 1000 days of a child's life (Grace et al. 2018; Lambrecht et al. 2023). However, livestock‐based interventions also have limitations. There is limited evidence to support interventions of only training or BCC without an accompanying livestock transfer (Janzen et al. 2018). Livestock interventions also raise questions of environmental sustainability (Mehrabi et al. 2020). Poultry interventions pose concerns for child morbidity from exposure to chicken feces, which is a subject of ongoing investigation (Passarelli et al. 2020).

While the evidence on the effectiveness of nutrition‐sensitive agriculture programs is growing, we know little about the costs of implementing these complex, integrated interventions (Ruel and Alderman 2013). A lack of standardized economic evaluation methods for multisectoral nutrition‐sensitive programs further hampers our understanding of the costs of implementation and complicates intervention comparisons (Gyles et al. 2012; Njuguna, Berkley, and Jemutai 2020; Ramponi, Tafesse, and Griffin 2021).

SEEMS‐Nutrition (Standardizing Economic Evaluation for Multisectoral Strategies for Nutrition) is a research consortium that developed a standardized methodology for economic evaluations of multisectoral nutrition‐sensitive interventions. The SEEMS‐Nutrition approach has been applied to nutrition‐sensitive interventions to build a body of evidence that is consistent, comparable and comprehensive. Establishing evidence on the costs and benefits of multisectoral nutrition programs is critical for policy decision‐making, encouraging government partnerships and continued donor investment.

This study applies the SEEMS‐Nutrition approach to the Soutenir l'Exploitation Familiale pour Lancer l'Élevage des Volailles et Valoriser l'Économie Rurale (SELEVER) project in Burkina Faso. SELEVER was designed to strengthen poultry value chains through a market‐facilitation approach that included BCC on nutrition and poultry income‐generation activities while promoting women's empowerment. SELEVER aimed to improve the health, diets and nutrition status of women and children through improved nutrition knowledge and agricultural/livestock practices. SELEVER's implementation was led by the NGO Tanager in partnership with several Burkinabe NGOs.

The purpose of this study is to measure the total incremental costs and costs per beneficiary of delivering the SELEVER intervention. The SEEMS‐Nutrition team collaborated with the International Food Policy Research Institute (IFPRI) on this research. IFPRI conducted a 5‐year impact evaluation of the SELEVER program. Details of the IFPRI SELEVER trial are published elsewhere (Becquey et al. 2022; Gelli et al. 2017; Gelli, Pedehombga, et al. 2019; Gelli, Headey, et al. 2019; Leight, Awonon, and Pedehombga 2022a, 2022b; Heckert et al. 2023). Briefly, SELEVER had modest effects on households' poultry production in larger producers, increasing their input use and reducing poultry mortality. In relation to diet quality during the lean season, children in SELEVER intervention groups were more likely to consume eggs. Finally, integrating a livestock BCC with water, sanitation and hygiene (WASH) component alongside the SELEVER poultry and nutrition intervention increased knowledge of livestock‐related hygiene risks and improved livestock hygiene‐related practices (Gelli et al. 2023).

In this study, we calculated the financial and economic costs of the SELEVER intervention including total incremental costs and unit costs. Financial costs are the implementer's actual expenditures on goods and services purchased to carry out the intervention. Economic costs are the opportunity costs of all resources used to reach project outputs and outcomes and can include the value of resources not paid for; in this case, volunteer time or beneficiary time. Costs were allocated to project inputs and activities to reveal the major cost drivers of the program. We also estimated the costs of integration across sectors and actors, as multisectoral programs typically involve several implementing partners.

### 1.1 The SELEVER Intervention

**Figure 1:**
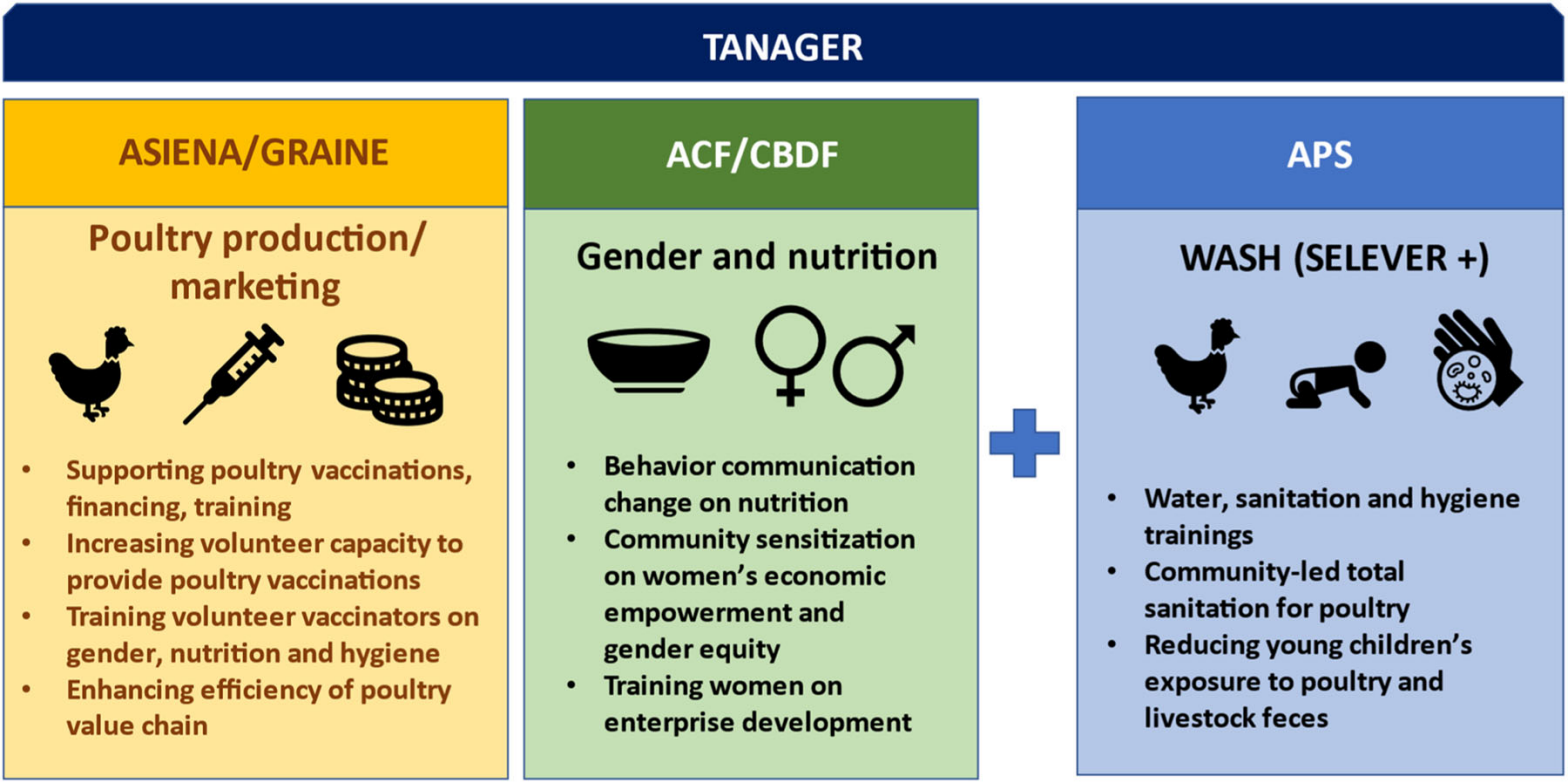
SELEVER intervention design and implementation (*Source:* Authors).

The Family Poultry Program to Improve Income and Nutrition (SELEVER) project was funded by the Bill and Melinda Gates Foundation and implemented over 5 years in Burkina Faso (Supporting Information S1: Appendix). The project was implemented by local NGOs led by Tanager with private institutions and the Government of Burkina Faso. In each region, NGOs provided overlapping activities within the project communities but did not necessarily coordinate with the same actors. In this ‘co‐location’ model, each NGO acted as an individual agent promoting sector‐specific activities targeted to different groups, with activities involving low levels of coordination across sectors. The NGOs implementing sectoral activities, including poultry, nutrition and gender, are outlined in the Supporting Information S1: Appendix Tables 1–3. SELEVER's implementation involved three components: (1) poultry production and marketing systems; (2) nutrition and gender; and (3) poultry production with water, sanitation and hygiene (WASH) BCC (Figure 1).

The first component of the project included support to poultry production and marketing based on access to financing, poultry vaccination and trainings on topics like poultry flock management. There were multiple groups involved in each village, each with a designated president and treasurer. Other key actors included village volunteer vaccinators (VVVs), who received additional training on poultry husbandry, including vaccinations as well as improved gender and nutrition practices. Once trained, VVVs provided paid services to the community, including vaccinations and deworming. Government extension workers (Direction Régionale de Agriculture et des Aménagements Hydrauliques ‐ DRRAH) provided additional VVV training and monitoring and supported NGO facilitators with training, market facilitation and vaccines/input provision.

The second and third components integrated nutrition BCC with gender sensitization and training, implemented at community‐level by local NGOs through trainings, peer group support and home visits. Nutrition BCC included materials and training delivered through poultry producer and women's groups. Community leaders also delivered nutrition BCC. Nutrition BCC topics included the promotion of nutritious diets, including IYCF. Gender sensitization activities included support for women's groups, promotion of gender equity and women's empowerment.

The fourth component supported poultry‐related WASH BCC, which was only implemented in one of the intervention arms (SELEVER‐WASH villages) to enhance impacts by reducing the risk of young child exposure to poultry feces. This intervention arm was based on a Community‐Led Total Sanitation (CLTS) approach (Crocker et al. 2017), and was adapted with materials that aimed to reduce poultry fecal exposure in partnership with the local NGO APS. APS also trained facilitators in CLTS and organized village hygiene committees.

The SELEVER program impact pathways are detailed in the protocol and process evaluation documents (Gelli et al. 2017; Gelli, Pedehombga, et al. 2019; Gelli, Headey, et al. 2019). The program has three impact pathways: (1) increasing demand for nutritious foods, (2) increasing the supply of nutritious foods, and (3) empowering women (Supporting Information S1: Appendix Table 4). Briefly, the first impact pathway aimed to increase intake of nutritious foods promoted by nutrition BCC. Households consume nutritious foods—including poultry products they produce—or purchase them. These actions stimulate demand for nutritious foods across the value chain, expanding poultry markets. Likewise, increased demand can in turn, stimulate supply. The second impact pathway targets the poultry value chain, supporting poultry producers in addressing market demand. Supply‐side activities included input provision for poultry production and poultry management training like promoting vaccination to reduce poultry mortality and to increase poultry producer incomes, improving consumption and demand for nutritious foods. The third impact pathway targeted women's empowerment. Gender activities included engaging with women's producer groups to support income generation from poultry and linking to savings and loan groups to improve access to credit. Gender sensitization also targeted village leaders and men.

## 2 Methods

We calculated the incremental financial and economic costs of an integrated poultry‐based agriculture‐nutrition intervention. All costs incurred by implementers and participants are included. External research costs such as program evaluation were excluded. This study applied the standardized SEEMS‐Nutrition approach to address the evidence gap on costing multisectoral nutrition programs (Levin et al. 2023). The SEEMS‐Nutrition approach includes a protocol template, data collection tools and cost allocation guidance and standardized activity and input categories. This analysis follows the Global Health Cost Consortium (GHCC) Reference Case for Estimating the Costs of Global Health Services and Interventions best practices guidance (Vassall et al. 2017).

The SEEMS approach has four steps that align intervention costs to a multisectoral nutrition typology. This approach identifies all intervention costs: both financial and opportunity costs. This study was conducted from the societal and payer perspectives. Standardized and unit costs are calculated by identifying resources and outputs along the program impact pathways. The first step of the SEEMS approach aligns the intervention to a typology inspired by the Scaling Up Nutrition (SUN) Compendium of Actions for Nutrition and by a typology of nutrition‐sensitive value (NSV) chains. NSV interventions augment and leverage opportunities to increase nutritional value along the supply chain for smallholder farmers. The NSV typology categorizes interventions that support the improvement of nutritional value along supply chains and provides a framework for comparison with other interventions (De la Pena, Garrett, and Gelli 2018). This NSV typology classifies interventions as (a) increasing demand for nutritious foods; (b) increasing supply of nutritious foods and (c) promoting the enabling environment for nutrition. An example of a demand‐side intervention component is nutrition‐focused BCC to increase consumption of nutritious foods, a supply‐side component is poultry extension, and the enabling environmentcomponent supports women's empowerment. Second, we link program impact pathways to the intervention outcomes and outputs with the NSV typology (Supporting Information S1: Appendix Table 5). Step three identifies inputs, activities and costs along these impact pathways. The fourth step combines total incremental cost estimates with program outputs to derive unit costs (Supporting Information S1: Appendix Figure 1).

SEEMS‐Nutrition provides standardized activity and input cost categories, as well as their definitions and guidelines (Supporting Information S1: Appendix Table 6). The SELEVER intervention activities and inputs were mapped to the SEEMS standardized categories (Supporting Information S1: Appendix Tables 7 and 8).

### 2.1 Data Collection

Costing data collection occurred at two points during the project: mid‐point and endline. Resource use was measured using financial expenditure data from Tanager—the primary grantee—and implementing NGOs, combined with micro‐costing to allow for estimation of both financial and economic costs. We identified activity cost centers and used a combination of project expense reports, financial and administrative records and qualitative data on time allocation and out‐of‐pocket (OOP) expenses to value inputs for each activity. We obtained full aggregate expenditures for all partners from Tanager financial reports. However, because not all the implementing NGOs included a full breakdown of expenditures, we could not calculate cost shares by activity and input directly for all NGOs. We undertook in‐depth analysis of complete expenditure data, where possible, and used it to obtain estimates of shares by input and activity. These estimates were then applied as assumptions in cases where full expenditure breakdowns were not available.

We conducted both quantitative and qualitative primary data collection for estimating resource use and costs, including: (1) project expenditures extracted from expense reports; (2) semi‐structured in‐depth interviews (IDIs), focus group discussions (FGDs), structured questionnaires and observation of staff, frontline workers and participant project activities. Quantitative costs were gathered from expenditures and project documents. Qualitative data collection included interviews with staff, government stakeholders, frontline workers and participants to capture details of their time use, and self‐reported contributions to project activities (Supporting Information S1: Appendix Table 9), in addition to the perceived benefits of participation in the intervention. Interview, FGD guides and accompanying consent forms were translated into French. Qualitative data was collected and transcribed by a French‐speaking collaborating researcher based in Burkina Faso. Quantitative estimates were extracted into Excel while complementary qualitative data was analyzed. Qualitative data informed time and resource allocation for time use of personnel, volunteers and others interviewed. Project field staff were also observed conducting training, distributing educational materials and performing other duties. We also reviewed planning and progress reports for randomly selected implementers and frontline workers. Data obtained from interviews was used to estimate averages and ranges of project‐related time use by each type of Tanager personnel, volunteer implementing partner and for project participants.

The data were entered into Microsoft Excel on secure password‐protected computers and servers and were stored in accordance with IFPRI and Tanager data policies. Data were accessible to the research team only. The anonymized electronic data were also stored on a University of Washington (UW) Secure Server. All hard copies of consent forms, structured interviews and timesheets were kept in a safe location accessible only to the research team. Documentation with identification was stored in a separate location.

Ethical clearance for the SELEVER impact evaluation and costing study was obtained from the IFPRI IRB in Washington, DC (approved 26/12/2016, ref: IRB00007490) and the Comité de Recherche en Santé MS/MRSI in Burkina Faso (N°2016‐12‐142). The trial was registered on the ISRCTN registry (ISRCTN16686478).

### 2.2 Data Analysis

Administrative financial data were entered into Excel and was assigned input cost category codes (Supporting Information S1: Appendix Table 8). Interview data were used to estimate averages and ranges of project‐related time use by implementing partners (Tanager, NGOs, volunteers) and participants. The number of household members reached was estimated using the number of participating households and the mean number of household members (8.7 members, a figure obtained from the program evaluation household survey).

We estimated the total government, volunteer and beneficiary time and valued it with either the appropriate civil servant cost per minute (Tanager) or the prevailing wage rate for rural or urban volunteers and participants. Labor and supply costs were allocated to activities and combined with financial expenditure data to obtain total financial and economic costs.

An analyst coded expenditure data in SEEMS‐Nutrition standardized Excel templates. One set of data entry sheets was used for administrative expense data for financial costs, while others captured data from qualitative interviews to calculate economic costs.

#### 2.2.1 Personnel Costs

Financial and economic costs related to program personnel were allocated across SELEVER activities. We combined time allocation data from qualitative primary data collection with program expenditures. NGO staff and VVV time use were gathered through IDIs and FGDs and this data informed how labor and supply costs were coded and mapped to a standardized activity cost category.

#### 2.2.2 Economic Costs

Estimates of the incremental costs of frontline VVVs—who are paid by farmers who hire them for services—include actual personnel costs obtained from estimates of OOP expenditures and time valuation. These economic costs were included as OOPs and for VVV time and travel. VVV profits from vaccinations and other services were subtracted from economic costs incurred.

#### 2.2.3 Beneficiary Opportunity Costs

Participants engaged in various activities, like group and community meetings, trainings and home visits. The opportunity costs of beneficiary participation were calculated using data from an IFPRI process evaluation (number and frequency of sessions attended, session duration, and number of participants). We gathered OOPs and average time per year spent on program activities. National mean agricultural wage rates were used to value beneficiary time. Personnel and beneficiary costs were mapped to SEEMS activity categories (Supporting Information S1: Appendix Table 7).

#### 2.2.4 Start‐Up and Capital Costs

Start‐up costs including capital and equipment for durable goods lasting over 1 year and valued at over USD$100 were annuitized over the 5‐year implementation. This annuitization assumed a discount rate of 3% and an expected useful life of 10 years, ensuring the estimation of the equivalent annual cost. Annuitization reflects the value in‐use of capital instead of the financial cost at time of purchase (Brooker et al. 2008). VAT taxes included in the commodity cost and durable goods taxes were incorporated into financial costing. Excluding small supplies, these taxes were not included in the economic costs. Costs are inflation‐adjusted and presented in 2020 $USD with an exchange rate of $1USD/567 CFA (West African Franc, XOF).^1^

#### 2.2.5 Unit Costs

Total incremental costs were calculated for direct program participants and for participating households. Program monitoring data provided the total number of participants (*n* = 86,150) and households (22,712) reached. The incremental beneficiary cost was calculated by the total cost divided by the number of direct participants. Total incremental costs were disaggregated by financial and economic components.

### 2.3 Sensitivity Analyses

We conducted a probabilistic sensitivity analysis using Monte‐Carlo simulations in the Crystal Ball software^2^ to assess uncertainty in the input estimates on total and unit costs. As the intervention depended on beneficiary group participation—in credit, gender and nutrition groups and village sanitation committees—we selected input parameters that reflected the importance of group participation (attendance). However, it is common that the intensity of group participation and attendance is variable in terms of frequency of group meetings. We assume that there would be higher participation from credit groups, as participation was a prerequisite for loans. However, attendance for other groups (e.g., nutrition and gender) had less stringent requirements.

Crystal ball assigns a distribution to assumptions from a base case. Based on the mean and SD, a normal distribution was assigned. We varied parameters: (1) average number of poultry meetings over 12 months; (2) average number of nutrition and gender meetings over 12 months; and (3) group meeting attendance rates from a base case of 0.75, and then varying this base case. We conducted 5,000 Monte‐Carlo simulations and varied these assumptions to understand which variables most influenced total and unit costs. We varied meeting intensity to understand cost variations under low‐, medium‐ and high‐intensity scenarios.

## 3 Results

Total incremental costs over 5 years were USD$18,084,727 (Table 1). Financial costs were USD$7,693,722, accounting for 43% of total costs. Economic costs were USD$10,391,503 (57%). Start‐up costs represented USD$1,558,292 (9% total costs). Recurrent costs were USD$16,526,435 (91% total costs).

**Table 1:** Summary of total incremental costs for the SELEVER intervention in $USD, 2019

	Economic	Financial	Total	%
Inputs				
Personnel	4,128,380.68	5,021,218.28	9,149,598.96	50.59
Agricultural input supplies	1,825,606.62	15,200.05	1,840,806.66	10.18
Equipment	0.00	555,821.58	555,821.58	3.07
Contracted services	0.00	853,825.96	853,825.96	4.72
Transportation	206,645.04	792,044.78	998,689.81	5.52
Travel/per diem/allowances	0.00	765,482.37	765,482.37	4.23
Other supplies	1,533,090.00	796,343.51	2,329,433.51	12.88
Overhead	0.00	1,591,068.83	1,591,068.83	8.8
Stage				
Start‐up	553,607.79	1,004,684.87	1,558,292.67	9
Recurrent	7,140,114.54	9,386,320.48	16,526,435.02	91
Activity				
Planning/microplanning	$0.00	$403,775.98	$403,775.98	2.23
Program installation	$0.00	$611,185.45	$611,185.45	3.38
Awareness raising/sensitization	$0.00	$136,444.54	$136,444.54	0.75
NGO recruitment	$0.00	$822,446.18	$822,446.18	4.55
Training	$584,139.01	$2,255,939.69	$2,840,078.70	15.70
Materials development	$0.00	$401,516.18	$401,516.18	2.22
Management	$0.00	$941,248.48	$941,248.48	5.20
Monitoring and evaluation	$0.00	$662,457.56	$662,457.56	3.66
Procurement	$0.00	$222,118.33	$222,118.33	1.23
Distribution of inputs	$0.00	$48,133.48	$48,133.48	0.27
Site supervision	$31,095.06	$0.00	$31,095.06	0.17
Home visits: household counseling	$801,440.91	$489,426.02	$1,290,866.94	7.14
Home visits: agriculture/poultry extension	$2,994,351.27	$71,801.33	$3,066,152.60	16.95
Community events/extension	$0.00	$103,761.07	$103,761.07	0.57
Establishing and running community groups	$0.00	$711,201.88	$711,201.88	3.93
Technical assistance	$1,144,527.97	$29,583.15	$1,174,111.12	6.49
Microcredit activities	$621,122.14	$527,041.52	$1,148,163.66	6.35
Volunteer recruitment	$0.00	$186,521.66	$186,521.66	1.03
Integration and coordination	$0.00	$0.00	$0.00	0.00
Overhead/indirect	$1,517,045.96	$1,766,901.41	$3,283,947.37	18.16
Total	$7,693,722.33	$10,391,503.90	$18,085,226.23	100.00

### 3.1 Cost Drivers

We disaggregate costs by activity and input type to understand the primary cost drivers. This process identifies how and where program resources are being directed. It also highlighsts where costs can be reduced, which is of interest to implementers. Cost drivers by program activity type are shown in Supporting Information S1: Appendix Figure 3. Other than overhead and indirect costs (18%), the main drivers of cost by activity were home visits for agriculture and poultry extension (17%), training (16%), home visits for household counseling (7%), technical assistance (6.5%), microcredit activities (6%), NGO recruitment (5%), establishing and running community groups (4%), monitoring and evaluation (4%), program installation (3%), planning (2%), materials development (2%), procurement (1%), volunteer recruitment (1%), awareness raising and sensitization (< 1%), community events (< 1%) and input distribution (< 1%) (Supporting Information S1: Appendix Figure 2). Costs by input type were driven by personnel (51%), other supplies (13%), agricultural inputs (10%), overhead (9%), transportation (6%), contracted services (5%), travel/per diems (4%) and equipment (3%) (not shown). The intervention was primarily a supply‐side driven intervention (47% of costs aligned to this pathway), as seen in the cost breakdown by NSV typology (Supporting Information S1: Appendix Figure 3). Costs were also examined by sector to reflect the multisectoral nature of the intervention. Most costs were inter‐sectoral (40%), with 23% of costs associated with agriculture and poultry, 22% with gender, but only 15% of costs related to nutrition.

### 3.2 Unit Costs

There were 86,150 total direct participants in the program (22,712 households with 197,594 members). The total direct beneficiary cost was USD$210. Total unit costs per household were $796 and the unit cost per household member was $92 (Table 2).

**Table 2:** Unit costs.

Unit cost measure	Economic cost	Financial cost	Total costs
Unit cost per household	$338.75	$457.53	$796.26
Unit cost per direct beneficiary	$89.31	$120.62	$209.92
Unit cost per household (all household members)	$38.94	$52.59	$91.52

### 3.3 Sensitivity Analysis

**Figure 2:**
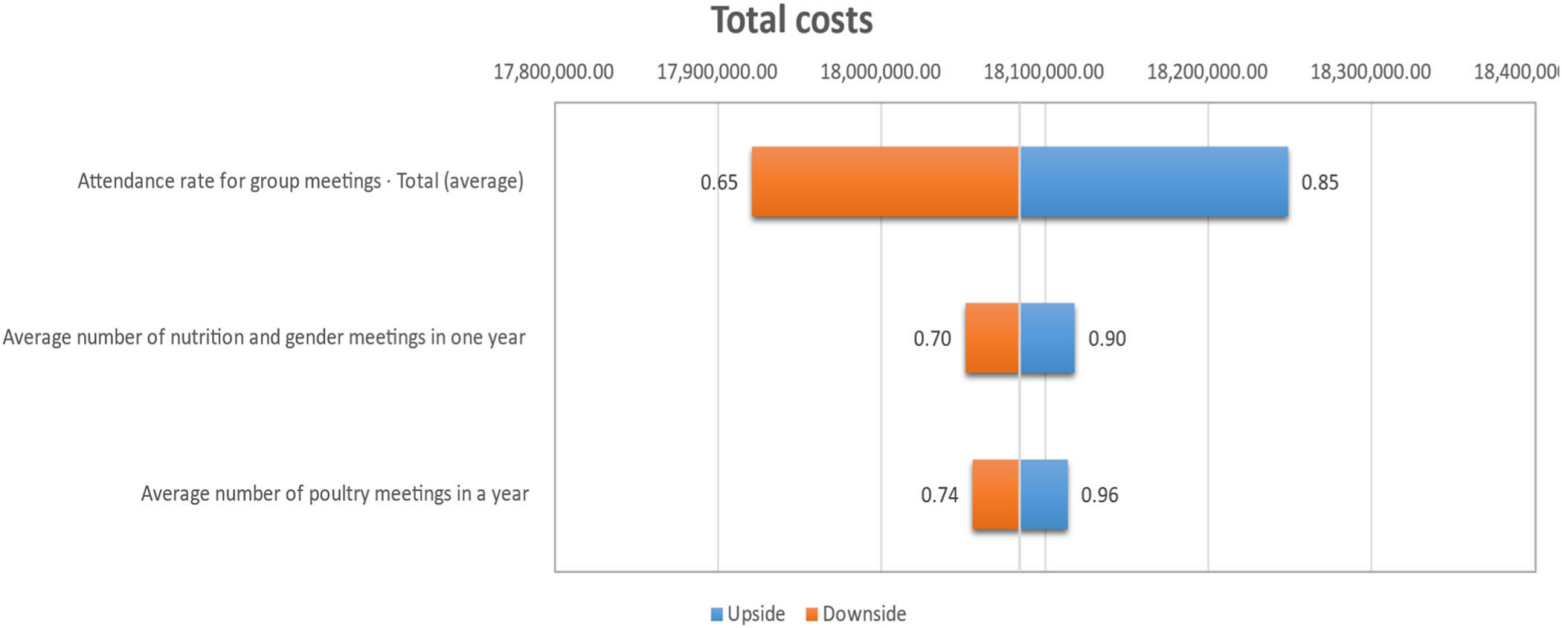
Tornado plot for sensitivity analysis (*Source:* Authors).

The sensitivity analysis shows variation in economic costs based on meeting intensity and attendance (Figure 2). The base case for group meeting attendance was 75%, ranging from 65% to 85%, to capture variation in group adherence and participation.

The low‐intensity scenario—representing how the program was implemented in practice—was measured as 0.85 nutrition and gender and 0.8 poultry meetings per year. Cost per beneficiary quickly and substantially increased with meeting frequency ‐ from $210 in the low intensity to $287 in the high‐intensity scenario (Table 3).

**Table 3:** Sensitivity analysis by meeting intensity.

Sensitivity analysis varying meeting intensity	Total cost	Cost per beneficiary (USD)	Cost per household (USD)	Cost per household member (USD)
Low intensity (current average)	$18,084,727.68	$209.92	$796.26	$91.52
Medium intensity (1 training each for poultry and nutrition and gender per quarter)	$19,968,174.40	$231.78	$879.19	$101.06
High intensity (1 training for poultry and nutrition and gender per month)	$24,710,040.04	$286.83	$1087.97	$125.05

*Source:* Authors.

## 4 Discussion

Multisectoral nutrition programs in LMICs aim to improve diets and nutrition in vulnerable populations. Despite their potential to address maternal and child nutrition, they lack detailed cost evidence. The SELEVER intervention in rural Burkina Faso sought to improve poultry production and the diets and nutritional status of women and children (Gelli, Pedehombga, et al. 2019; Gelli, Headey, et al. 2019). This study addresses the gap by presenting total incremental costs, financial and economic costs and per‐beneficiary costs SELEVER, contributing valuable evidence to support the design and scaling of similar nutrition‐sensitive programs.

We present the unit costs and costs disaggregated by activity and input type to explore the primary cost drivers of the program. Total unit costs per direct beneficiary ($210) were above the upper range of other nutrition‐sensitive interventions evaluated with the same standardized costing approach ($132 in Nepal, $157 in Bangladesh, $160 in Malawi) (Margolies et al. 2021; Thai et al. 2023). Also, in these other interventions, costs were primarily driven by financial—rather than economic—costs; 75% (Malawi), 76% (Bangladesh) and 53% (Nepal) (Choo et al. 2024). In contrast, in the SELEVER program in Burkina Faso, only 43% of costs were financial, and over 50% of the costs were economic costs. To explore the SELEVER program structure on participant time and opportunity costs, the sensitivity analysis was used to vary meeting intensity and attendance. Higher intensity quickly increased cost per beneficiary, reflecting their opportunity cost to attend meetings (ranging from $209.92 in the low intensity scenario to $286.83 in the high‐intensity scenario). These estimates suggest that the SELEVER co‐location model contributed to higher beneficiary opportunity costs since participants spent more time attending multiple groups running in parallel. In other words, due to the co‐location model, participants had a greater number of meetings to attend, rather than attending coordinated sessions that integrated gender, nutrition and livestock messages. These relatively high‐opportunity costs may have posed important barriers both in terms of program uptake and potential sustainability of program activities. Additionally, participants spent significant time in home visits (17% of costs) and trainings (16%). So, although the program objectives were multisectoral in nature, the program activities ran in parallel. Program activities were not designed to be integrated in order to maximize coordinated messaging across sectors and to minimize their impacts on participants' time.

Examining costs by activity, a fifth of costs were allocated to essential overhead and indirect activities. In contrast, in the Bangladesh intervntion, only 4% of costs were for overhead (Thai et al. 2023). These costs are similar to a multisectoral intervention in Nepal (16%)—a scaled‐up project that also had a large scale like SELEVER (Choo et al. 2024). This finding likely reflects SELEVER's three‐level organizational setup: a single, top‐level, international NGO providing administrative and technical oversight, with multiple local implementing NGOs at the second level, and a third level of community groups. This multilevel structure was necessary to provide technical assistance and to fill local capacity gaps. SELEVER was supply‐side oriented, reflected in the allocation of costs (47% to supply‐side components). Another agriculture‐nutrition intervention based on a microcredit platform in Bangladesh costed under SEEMS‐Nutrition and aligned to the same NSV typology showed nearly half of costs were associated with demand‐side components, 23% to supply‐side and a third to the enabling environment (Thai et al. 2023). Similarly, in an agriculture‐nutrition intervention in Malawi costed with SEEMS‐Nutrition, half of costs were incurred by demand‐side components (Margolies et al. 2021). In Nepal's scaled‐up multisectoral nutrition program, the majority of costs were associated with demand‐side components and only 16% went to supporting supply‐side actions (Choo et al. 2024). SELEVER had a lower percentage of costs associated with generating demand for nutritious foods (31%) than the programs in Nepal (62%), Bangladesh (47%) and Malawi (53%), but it had similar cost shares to support the enabling environment (20%).

It is important to consider intervention costs with intended impacts. The SELEVER impact evaluation showed it did not significantly impact the primary dietary outcomes—probability of adequacy (PA) of zinc, Vitamin A, mean PA of 11 micronutrients and dietary diversity scores for women and index children (2–4 years) and minimum acceptable diets in younger siblings (6–23 months) (Becquey et al. 2022). However, SELEVER increased lean season women's PA of iron intake and index child egg consumption (Becquey et al. 2022). Only 15% of SELEVER costs by sector were associated with nutrition, and according to the NSV typology, only 31% of costs were associated with demand generation for nutritious foods—reflecting the intervention's supply‐side focus. Also, while 17% of costs were for agriculture and poultry extension, only 7% were for household counseling and 2% for BCC materials development. Again, this reflects the intervention's design to improve supply‐side poultry knowledge and practices. However, SELEVER did not increase average poultry flock sizes in the study population, although it did in the larger producer subgroup, which also improved poultry knowledge and practices (Leight, Awonon, and Pedehombga 2022a). On average, intervention households used more poultry inputs and generated higher revenues; however, their profits did not increase (Leight, Awonon, and Pedehombga 2022b).

The sensitivity analyses showed that increases in meeting intensity can significantly inflate costs, raising questions about the implementation intensity of the intervention. While increasing the intensity of meetings for activities such as poultry extension, gender and nutrition raise unit costs, greater implementation strength could improve outcomes. For example, increased exposure to gender, nutrition and poultry extension messages could influence beneficiary behaviors and practices related to diets, nutrition and poultry production. Other potential benefits of higher intensity implementation could include the reinforcement of social support aspects of group membership through more regular group contact. However, these conclusions cannot be reached without supporting data to evaluate alternative scenarios. Understanding the cost trade‐offs of program intensity can aid implementers in decision‐making around design. The the second iteration of SELEVER now underway. SELEVER2 departs from co‐location to a fully integrated model of integrated components, strengthening coordination of community activities and potentially lowering costs and enhancing effectiveness. This would increase intensity of implementation but also lower the number of participants—but also lower opportunity costs. This integrated approach remains an important area of ongoing research. The SEEMS‐Nutrition methodology will be applied to more economic evaluations of multisectoral nutrition programs, building the evidence comparing interventions with diverse designs and platforms.

## 5 Conclusions

This study presents the total incremental costs of a multisectoral nutrition program implemented through a poultry platform. Estimates of costs per beneficiary were high compared to evidence of the costs of other multisectoral nutrition‐sensitive interventions—particularly in terms of beneficiary opportunity costs. Opportunities exist for cost containment, including modifications that could enhance efficiency and effectiveness. Applying a standardized approach to costing facilitates comparison of diverse multisectoral nutrition interventions delivered through different platforms, facilitating decision‐making by governments, donors and nonprofits.

## Author Contributions

Amy Margolies contributed to the cost study methodology, cost analysis and led the drafting of the manuscript. Abdoulaye Pedehombga led the data collection. Aisha Twalibu led the financial cost analysis. Odiche Nwabuikwu contributed to the cost analyses. Jolene Wun contributed to the conception and design and financial cost analysis. Chris Kemp contributed to the conception and design. Aulo Gelli led the conception and design, economic and cost analysis and contributed to the cost study methodology, financial cost analysis and manuscript. Carol Levin contributed to the conception and design and cost study methodology, data collection and manuscript and provided technical advice. All authors reviewed and approved the final manuscript.

## Conflicts of Interest

The authors declare no conflicts of interest.

## Supporting information

Supporting information.

## Data Availability

The data that support the findings of this study are available from the corresponding author upon reasonable request.

## Endnotes

1
*Source*: https://www.xe.com/currencyconverter/convert/?Amount=1&From=USD&To=XOF, November 2019.

2 Crystal Ball software, Oracle. https://www.oracle.com/applications/crystalball
