# Supplementary material for: The Costs of a Multisectoral Nutrition Program Implemented Through a Poultry Value Chain Platform in Burkina Faso
Source: Matern Child Nutr. 2025 Jan 3;21(2):e13791. doi: 10.1111/mcn.13791 (PMC11956039; doi:10.1111/mcn.13791)
Supplement: Supplementary file 1 — Supporting information. [file MCN-21-e13791-s001.docx]

**Appendix**

**Appendix Table 1. Role and responsibilities of NGO actors involved in the SELEVER poultry-related activities**

| **Organisation** | **Actors** | **Location** | **Role/Responsibilities** | **Remarks** |
| --- | --- | --- | --- | --- |
| **DRRAH** | Regional directors (3 regions) | Bobo, Koudougou, Dédougou | Management and administration | Government staff |
|  | SELEVER regional focal points | Bobo, Koudougou, Dédougou | Training facilitators, monitoring and evaluation | Government staff |
|  | Provincial directors | Provinces | Administration, training management | Government staff |
|  | Heads of Livestock zones | Communes | Training and monitoring at village level | Government staff? |
| **GRAINE** | Director/ coordinator | Ouagadougou | Management and administration | NGO staff |
|  | SELEVER project lead | Koudougou | Training facilitators, monitoring and evaluation | NGO staff |
|  | Facilitators (7 in total) | Communes | Training and monitoring at village level | NGO staff |
|  | GS, leaders, VVVs | Villages | Training and facilitation for participants | Volunteers |
| **ASIENA** | Director of operations | Ouagadougou | Management and administration | NGO staff |
|  | SELEVER project supervisor | Dédougou | Training facilitators, monitoring and evaluation | NGO staff |
|  | Facilitators (28 credit agents) | Communes | Training and monitoring at village level | NGO staff |
|  | MUSO, leaders, VVVs | Villages | Training and facilitation for participants | Volunteers |

**Appendix Table 2.** Role and responsibilities of actors involved in the SELEVER nutrition- and gender-related activities

| **Organisation** | **Role** | **Location** | **Responsibilities** | **Remarks** |
| --- | --- | --- | --- | --- |
| **CBDF** | Coordinator | Ouagadougou | Management and administration |  |
|  | SELEVER project lead | Koudougou | Training facilitators, monitoring and evaluation |  |
|  | Supervisors | Provinces (Léo, Koudougou, Bobo, Réo) | Training and monitoring |  |
|  | Facilitators (15 in total) | Communes | Training and monitoring at village level |  |
|  | GS, MUSO, leaders, model women, champion husbands | Villages | Training and facilitation for participants | Volunteers |
| **ACF** | Coordinator | Ouagadougou | Management and administration |  |
|  | SELEVER project lead | Dédougou | Training facilitators, monitoring and evaluation |  |
|  | Facilitators (12 in total) | Communes | Training and monitoring at village level |  |
|  | MUSO, leaders | Villages | Training and facilitation for participants | Volunteers |

**Appendix Table 3.** Role and responsibilities of actors involved in the SELEVER poultry-WASH activities

| **Organisation** | **Role** | **Location** | **Responsibilities** | **Remarks** |
| --- | --- | --- | --- | --- |
| **APS** | Director | Ouagadougou | Management, administration | NGO staff |
|  | WASH technical lead | Ouagadougou | WASH/CLTS Training | NGO staff |
|  | SELEVER project lead | Koudougou | Training and monitoring | NGO staff |
|  | Facilitators (10) | Province | Training WASH/CLTS at village level | NGO staff |
|  | Facilitators | Commune | Training, monitoring including home visits | NGO staff |
|  | Village level committees | Villages | Sensitisation, home visits | Volunteers |

**Appendix Table 4.** Program impact pathways for the SELEVER intervention^^[[1]](#footnote-1)^^

**
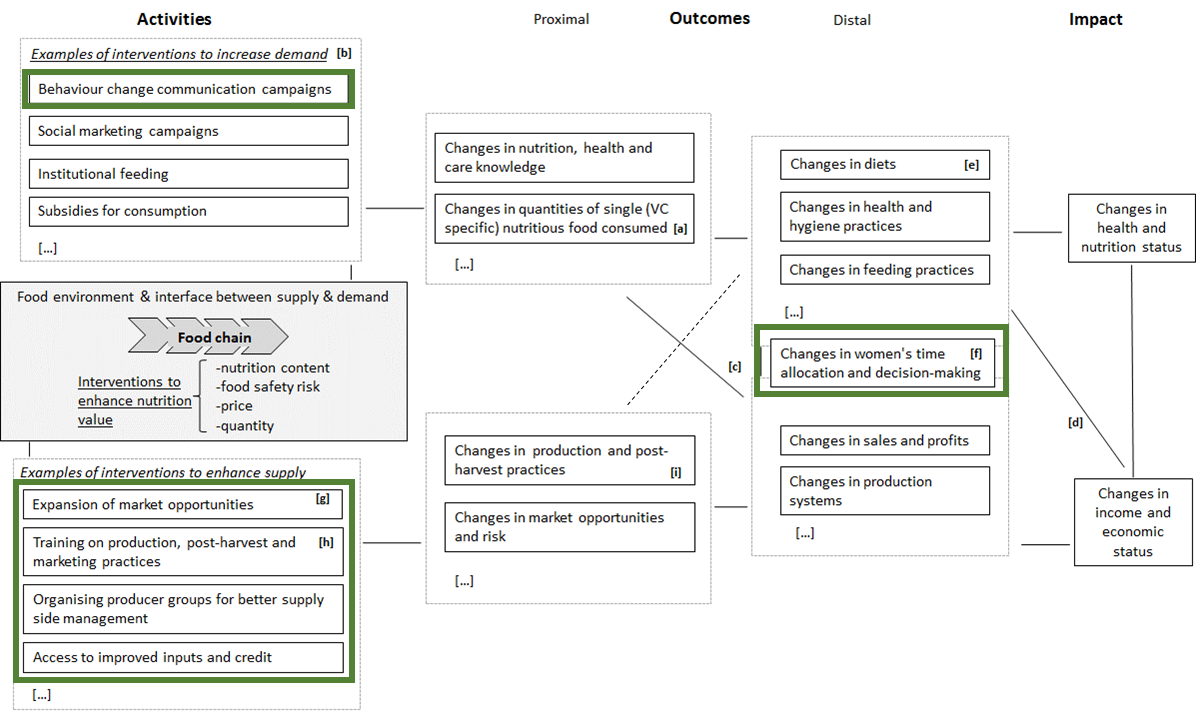
**

**Appendix Table 5.** Nutrition-sensitive Value (NSV) Chain Activity Coding

| NSV Intervention Typology | Entry Point | Activity |
| --- | --- | --- |
| Increase supply | Diversification/promotion of nutritious crops | - Training farmers on poultry production and marketing practices - Training volunteer vaccinators on vaccination services - Provision of inputs (poultry vaccinations) - Organizing producer groups for better supply side management |
| Increase demand | Behavior change communication | - Behavior change communication campaigns on nutrition, infant and young child feeding, hygiene and health through women’s and producer’s groups |
| Enabling Environment | Women’s Empowerment | - Community level sensitization on women’s economic empowerment and gender equity - Training women’s group participants on enterprise development, village savings and loans, child feeding |
| Shared program costs allocated to above three typologies | Coordination/Support | - Program management, administration |

**Appendix Figure 1.** Unit cost framework for SELEVER (Source: Authors)

**Appendix Table 6.** SEEMS standardized activity categories

| **Activity** | **Definition** |
| --- | --- |
| **START UP COSTS** |  |
| Awareness raising/sensitization | Awareness raising and sensitization at all levels, national government, regional, local, community |
| Materials development | Development of BCC and training materials |
| Monitoring and evaluation | Development of monitoring system |
| Planning/microplanning | Meetings, stakeholder meetings, assessments, situation analysis, program design |
| Procurement |  |
| Program installation | Establishing an office, NGO staff and frontline worker recruitment/hiring, soliciting bids and issuing contracts for contracted services, equipment purchases |
| Overhead |  |
| Training | Training at all levels:  NGO staff, Frontline workers, communities, individuals, this can be onetime only, or refresher training.  For coding purposes, always indicate if training/start up, or training recurrent, see below |
| Vendor/volunteer recruitment | Recruitment of community volunteers and vendors for market-based intervention |
| **RECURRENT COSTS** |  |
| Community events/extension |  |
| Distribution of inputs | Distribution of inputs to volunteers, vaccinators, households, individuals; may include supply chain costs |
| Establishing and running community groups | Includes demand generation activities to increase participation, related to establishment and regular running of groups (credit groups, women's groups, etc) |
| Home visits: household counseling | Primarily nutrition and health counselling |
| Indirect/overhead | Overhead/indirect includes communication, utilities, certain types of administrative personnel time, allowances, organizational indirects. |
| Integration and coordination | Recurrent and regular planning/coordination/  feedback meetings across all or some implementing partners/intentional cross-sectoral training.  Include regular planning and review meetings here. |
| Management |  |
| Microcredit activities | Loan disbursement and collection |
| Monitoring and evaluation | Designing and implementing regular monitoring and evaluation system; does not include impact or process evaluation activities |
| Planning/microplanning |  |
| Procurement | Procurement of inputs for volunteers, vaccinators, households, individuals |
| Technical assistance |  |
| Training | Recurrent training/refresher training |
| Site supervision | Supervision of front-line implementation and service delivery. Not financial oversight, not management of office staff. |

**Appendix Table 7.** SELEVER activities mapped to SEEMS standardized activity categories

| **Activity** | **Definition** |
| --- | --- |
| **START UP COSTS** |  |
| Awareness raising/sensitization | Awareness raising with NGO partners, government |
| Establishing and running community groups | Starting credit and women’s groups at village level |
| Management | Start-up management/administration for NGOs and at village level |
| Materials development | Development of BCC materials for trainings on nutrition and poultry production/marketing, production, and printing materials |
| Monitoring and evaluation | Situational analysis, assessments, landscape analysis, creation of monitoring system |
| Planning/microplanning | Orientation and kickoff workshops, village identification, start-up NGO administration |
| Procurement | Procurement of equipment and NGO partners |
| Program installation | Establishing office, NGO selection, NGO staff and frontline worker recruitment/hiring, establishing links with credit agency and loan guarantee |
| Overhead | Contracted services |
| Training | NGO training, training CLTS facilitators and village volunteers |
| Vendor/volunteer recruitment | Recruitment of community volunteers, vendors |
| **RECURRENT COSTS** |  |
| Community events/extension | Stakeholder engagement events, village triggering events, district government certification, ODF celebration |
| Distribution of inputs | VVV provision of vaccines, input support to producers |
| Establishing and running community groups | Women’s group meetings, credit group meetings for producers, meetings for natural leaders and/or WASH/CLTS committees |
| Home visits: household counseling | Household visits to group members, natural leaders perform home visits |
| Indirect/overhead | Communication, utilities, organizational indirects |
| Integration and coordination | Coordination with government |
| Management | Coordination/management of program and group operations |
| Microcredit activities | Loan disbursement and collection |
| Monitoring and evaluation | Village visits, leaders/committees monitor sanitation status, monitoring VVVs, credit use and households, program monitoring and evaluation |
| Planning/microplanning | Program and stakeholder meetings, program design, annual planning meetings, recruitment updates |
| Procurement | Acquisition of supplies (i.e., vaccines), supply and logistics for groups |
| Technical assistance | Technical assistance to support enterprises, vendors (distinct from training) |
| Training | Training women’s associations on enterprise development, nutrition training, staff development/training, VVV/community actor trainings |
| Site supervision | Supervision of implementation and service delivery |

**Appendix Table 8.** SELEVER inputs mapped to SEEMS standardized input categories

| **SEEMS Input Category** | **SELEVER Input Description** |
| --- | --- |
| **Agricultural supplies** | Vaccines |
| **Contracted services** | Workshop venue package |
| **Equipment** | Vehicles, bicycles, motorbikes purchased |
| **Other supplies** | Office supplies, purchased materials, community awards and celebration costs |
| **Overhead/indirect** | Office rent |
| **Personnel** | Paid staff time, unpaid time, hired labor |
| **Transportation** | Transportation, insurance, maintenance, rentals |
| **Travel/per diem/allowances** | Travel, per diems |

**Appendix Table 9.** Qualitative costing interview methods

| **Actor type** | **Interview type** | **Number of participants per interview** | **Average length of interview** | **Number of interviews** | **Total participants** |
| --- | --- | --- | --- | --- | --- |
| **Data Collection - Round 1 (2019)** | | | | | |
| Village volunteer vaccinators (VVV)- Poultry vaccinators | Individual in-depth interviews | 1 | 1 hour | 12 | 12 |
| Credit groups (Mutuelle de Solidarité (MUSO) et Groupe de Solidarité (GS) | Focus group discussions | 4 | 1 hour | 3 | 13 |
| Gender and nutrition groups | Focus group discussion | 4 | 1 h | 3 | 12 |
| Village and neighborhood Sanitation committee | Focus group discussions | 4 | 1 h | 3 | 13 |
| Agricultural extension officer | Individual in-depth interviews | 1 | 1 h | 6 | 6 |
| **Data Collection - Round 2 (2020)** | | | | | |
| Village volunteer vaccinators (VVV)- poultry vaccination | Individual interviews | 1 | 1 h | 13 | 13 |
| Credit Groups (Mutuelle de Solidarité (MUSO) et Groupe de Solidarité (GS) | Focus group discussions | 4 | 1 h | 6 | 23 |
| Gender and nutrition groups | Focus group discussions | 4 | 1 h | 6 | 24 |
| Village and neighborhood sanitation committee | Focus group discussions | 2 | 1 h | 5 | 10 |
| Agricultural extension officer | Individual in-depth interviews | 1 | 1 h | 6 | 6 |

**Appendix Figure 2. Cost drivers by activity type for the SELEVER intervention in Burkina Faso (Source: Authors)**

**Appendix Figure 3. Cost drivers mapped to the nutrition-sensitive value chain typology for the SELEVER intervention (Source: Authors)**

1. Boxes highlighted in green indicate SELEVER program impact pathways. [↑](#footnote-ref-1)
